# Bone mineral density and vertebral fractures in patients with systemic lupus erythematosus: A systematic review and meta-regression

**DOI:** 10.1371/journal.pone.0196113

**Published:** 2018-06-13

**Authors:** Claudia Mendoza-Pinto, Adriana Rojas-Villarraga, Nicolás Molano-González, Erick A. Jiménez-Herrera, María de la Luz León-Vázquez, Álvaro Montiel-Jarquín, Mario García-Carrasco, Ricard Cervera

**Affiliations:** 1 Systemic Autoimmune Diseases Research Unit, Hospital de Especialidades, UMAE CMNMAC—CIBIOR, Instituto Mexicano del Seguro Social, Puebla, Puebla, México; 2 Department of Immunology and Rheumatology, Medicine School, Benemérita Universidad Autónoma de Puebla, Puebla, Puebla, México; 3 Artmedica IPS, Bogotá, Colombia; 4 Center for Autoimmune Diseases Research (CREA), School of Medicine and Health Sciences, Universidad del Rosario, Bogotá, Colombia; 5 Research in Health Unit, UMAE, Instituto Mexicano del Seguro Social, México, Puebla, Puebla, México; 6 Department of Autoimmune Diseases, Hospital Clinic, Barcelona, Catalonia, Spain; Peking University First Hospital, CHINA

## Abstract

**Background:**

Observational studies have indicated a high but heterogeneous prevalence of low bone mineral density (BMD) and vertebral fractures (VF) in patients with systemic lupus erythematosus (SLE). Therefore, the objectives of this systematic review and meta-regression were: 1) to compare BMD between SLE patients and healthy controls and 2) to evaluate the relationship between BMD and glucocorticoid therapy and VF in SLE patients.

**Methods and findings:**

Articles were identified from electronic databases (PubMed, Embase, VHL, SciELO and the Cochrane Library). Prospective longitudinal and cross-sectional studies were considered for review. We evaluated the quality of the evidence included using the Oxford Centre for evidence-based medicine (EBM) Levels of Evidence. In total, 38 articles were identified and analyzed (3442 SLE cases and 6198 controls) in the analysis of BMD (9232 women and 408 men). There were significant differences in mean BMD between SLE patients and controls. BMD mean difference in cases/controls: -0.0566 95% CI (-0.071, -0.0439; p = < 0.0001). When only SLE patients were analyzed, the BMD did not significantly differ between patients who had or had not received glucocorticoid (GCT) therapy. 694 SLE patients were included in the analysis of VF (189 with VF vs. 505 without VF). Patients with VF had lower BMD than patients without VF (BMD mean difference without VF/with VF: 0.033 (95%CI: 0.006–0.060); p-value: 0.0156).

**Conclusions:**

Patients with SLE had lower BMD than healthy controls. Moreover, SLE patients with VF had lower BMD than patients without VF. However, our data did not show that GCT therapy had an impact on BMD.

## Background

Patients with systemic lupus erythematosus (SLE) may have an increased risk of bone mineral density (BMD) loss and vertebral fractures (VF) according to cross-sectional studies [1–4]. Osteoporosis and fractures contribute to damage in the musculoskeletal system, which is frequently involved in patients with SLE [5].

BMD measurement by dual energy X-ray absorptiometry (DXA) is the gold standard to assess fracture risk in healthy men and women [6]. The utility of DXA in predicting fracture risk in SLE is unclear for two reasons. First, only small cross-sectional studies have reported on the use of DXA to discriminate the fracture status. Secondly, the relationship between low BMD and glucocorticoid therapy (GCT), which is extensively used for the treatment of SLE disease flares and complications, remains unclear [2,4,7,8].

Identifying prevalent VF is important, since prevalent vertebral deformities are associated with a reduced quality of life [9], and increased mortality and risk of future fractures in the general population [10]. There are only a few studies on prevalent VF (using a standardized method of scoring vertebral deformities) and these showed at least one VF in 20–26.1% of SLE patients [2,11,12]. We recently showed that 20% of 110 SLE patients [median follow-up 8 (IQR 8–9) years] had radiographic VF at baseline and 32% had a new VF. The reported annual incidence rate of new morphometric VF is 3.5 (95% CI 2.4–4.91) per 100 patient/years [13]. A recent meta-analysis, including studies of VF prevalence and low BMD, reported an almost three-fold higher risk of VF in SLE patients (RR 2.97, 95% CI 1.71–5.16, *P* < 0.001) compared with healthy controls [14]. Another recent systematic review without a meta-analysis also concluded that SLE patients are at risk of both reduced vitamin D plasma levels and low BMD [15].

Of patients with VF, 29–35.8% had normal BMD [11,12], in line with results from studies in the general population reporting that the proportion of fractures attributable to osteoporosis is only 10–44%. This points to the limited value of BMD measurement in the assessment of future fracture risk. Moreover, these discrepancies between BMD and fracture risk may be because, in SLE, poor bone quality rather than decreased bone density plays the most important role in determining the risk of fractures, and VF occur at much high rates than expected on the basis of BMD, suggesting that the bone fragility of GCT users is not defined by the BMD. Indeed, cutoff values of BMD at the lumbar spine and femoral neck in women with VF treated with GCT were higher than those of controls [16].

Only one systematic meta-analysis has reported that SLE patients have significantly lower BMD levels than controls, and SLE is also significantly associated with increased fracture risk [14]. However, the relationship between BMD and GCT use and VF (using a standardized method of scoring vertebral deformities) has not been assessed in a meta-analysis or meta-regression. Therefore, we conducted a meta-analysis and meta-regression of studies evaluating: 1) BMD in SLE patients and controls, 2) BMD in SLE patients receiving GCT or not, and 3) BMD in SLE patients with or without VF.

## Materials and methods

### Search strategy

A systematic literature review was conducted using the following electronic databases: PubMed (1946-Week 2, January 2018), Cochrane library (1985-Week 2, Week 2, January 2018), EMBASE (1974-Week 2, January 2018), Virtual Health Library (VHL) (1998-Week 2, January 2018), and SciELO (1997-Week 2, January 2018), for published studies. We followed the PRISMA guidelines (Preferred Reporting Items for Systematic Reviews and Meta-Analysis, see S1 File) for meta-analysis of observational studies [17] in the data extraction, analysis, and reporting.

The following Medical Subject Heading (MeSH) terms were used: "Lupus Erythematosus, Systemic," "Osteoporosis," "Bone Density," "Densitometry” and "Spinal Fractures". Furthermore, we used ‘text words’ if there was no MeSH term, such as the cases of “SLE” abbreviation, “bone loss”, “BMD” abbreviation, “dual energy X ray absorptiometry”, “vertebral fractures” and “vertebral fragility”. DeCS terms (Health Sciences Descriptors) were also used to find records and sources of information through controlled concepts in order to search the SciElo and VHL databases. Studies were limited to those carried out in adult humans and published in English. References from the articles deemed relevant were hand-searched.

### Study selection and eligibility criteria

Prospective longitudinal and cross-sectional studies in SLE patients (regardless of menopausal status) were considered for review. Studies that used accepted and validated classification criteria for SLE were included. Case reports, conference abstracts, letters to editors, and studies not reporting the prevalence of osteoporosis or low BMD or VF prevalence were excluded. Papers were included if BMD was evaluated by dual-energy X-ray absorptiometry (DXA) at any of the total hip, femoral neck and lumbar spine. We included studies where VF was identified by standardized vertebral morphometry. In addition, the reference lists of relevant reviews and articles were manually retrieved to find other possible studies.

When various reports from the same study were published, only the most recent or informative one was included. However, if more than one publication described a single study but each presented new and complementary data, both were included and analyzed in separate analyses.

We first reviewed the titles and abstracts of studies found in the literature search and decided whether they conformed to the following research topics: 1) mean difference in BMD levels between SLE patients and controls, 2) mean difference in BMD levels between women and men with SLE, 3) mean BMD differences in patients with and without GCT, and 4) mean BMD differences in SLE patients with and without VF. Articles selected were evaluated by two investigators independently using the same selection criteria. The two resulting databases were compared and disagreements resolved by consensus.

### Data extraction

The following data were extracted from studies included: first author, publication year, study design, population studied, number of participants, number of cases and controls, mean age, ethnicity, use of GCT, cumulative GCT dose, BMD measurement sites and VF assessment method. Definitions of GCT therapy were taken from the articles included (S2 Table).

We were able to extract several effect sizes from a single study. For example, some studies reported mean BMD for cases and controls, disaggregated by region and gender. The nested nature of the data is taken into account in the multilevel linear (mixed-effects) model (see statistical methods).

### Assessment of methodological quality

The Oxford Centre for Evidence-Based Medicine (EBM): 2011 Levels of Evidence criteria were used to assess the strength of the evidence for all studied included studies [18]. Clinical evidence was divided into 5 levels ranging from I to V as follows (diagnosis question): Level 1 Systematic review of cross-sectional studies with consistently applied reference standard and blinding; Level 2 Individual cross-sectional studies with consistently applied reference standard and blinding; Level 3 Non-consecutive studies, or studies without consistently applied reference standards; Level 4 Case-control studies, or “poor or non-independent reference standard; Level 5 Mechanism-based reasoning. Any disagreement was resolved through discussion and consensus of investigators.

## Statistical methods

Due to the diversity of studies found, three meta-regression analyses were made: The first aimed to assess possible differences in BMD between SLE cases and controls. The second analyzed the effect of treatment (GCT) on BMD in SLE cases and the third evaluated differences in BMD between SLE cases with and without VF.

We were able to extract more than one effect measure from a single study; therefore, we fit a multilevel linear (mixed-effects) model [19] to the three scenarios. The first hierarchical level corresponds to a single effect measure, and in the second level several effect measures are reported.

The following strategy was employed in order to obtain a final, most parsimonious model in each case: first, the variance component structure was obtained by fitting several models with a saturated fixed-effects structure (all relevant covariates plus full interactions) and different variance models (fixed effects, mixed effects and multilevel mixed-effects). The most parsimonious variance structure was chosen according to the basis likelihood ratio test and AIC criteria. Once the variance structure was determined, the mean structure was assessed, subtracting interactions and covariates and, again, the most parsimonious mean structure was chosen on the basis likelihood ratio test and AIC criteria. Heterogeneity was calculated using Higgins’s (I2) tests. The I2 test showed the proportion of observed dispersion that was real rather than spurious and was expressed as a ratio ranging from 0% to 100%. I2 values of 25%, 50%, and 75% were qualitatively classified as low, moderate, and high respectively.

Using the selected models, the rank correlation test for funnel plot asymmetry was assessed to check for publication bias. The analysis was performed with the R 3.3.2 metaphor package [20].

## Results

### Identification of relevant studies

**Fig 1:**
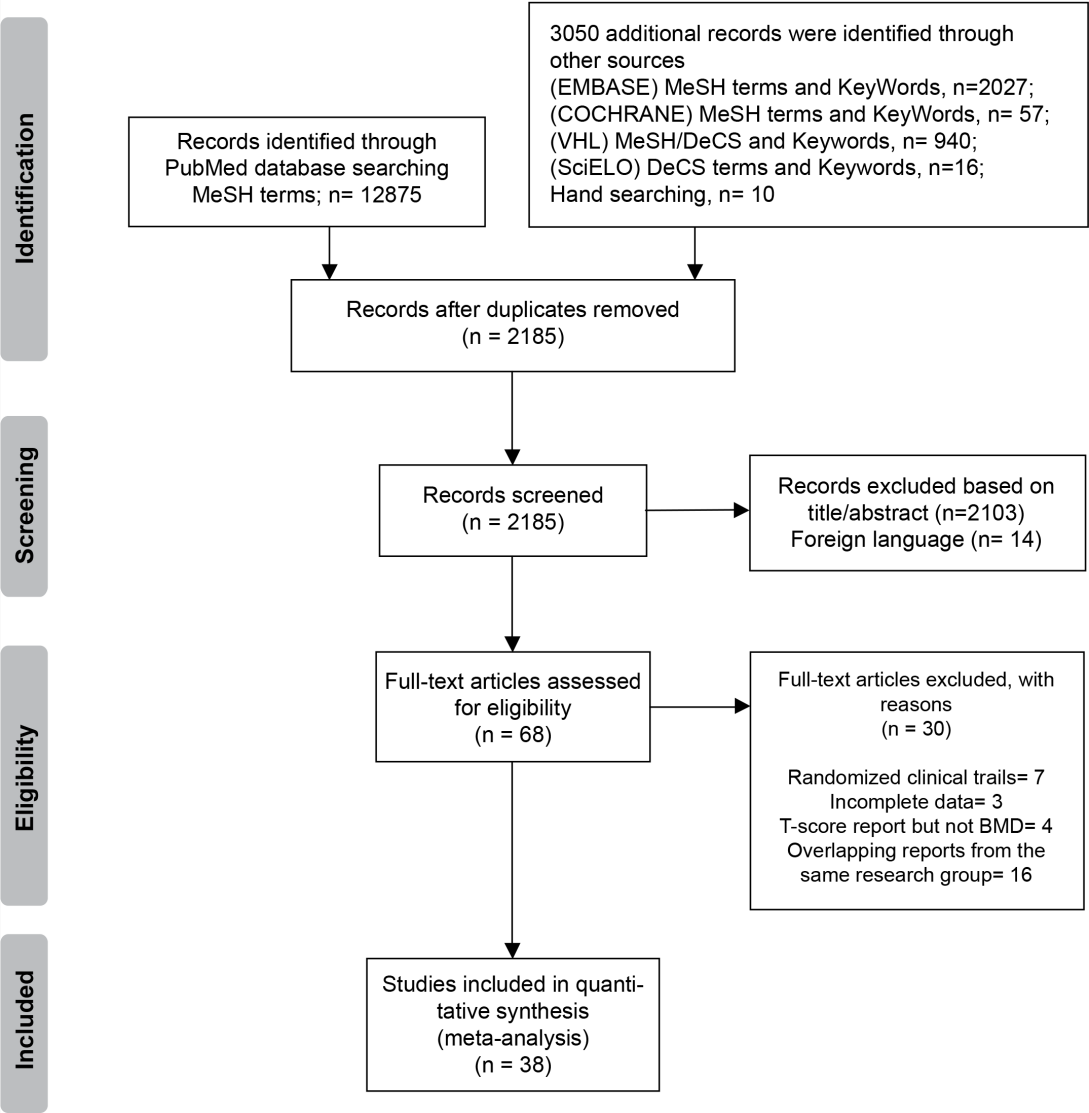
Flow diagram detailing the literature search.

Fig 1 shows a flow diagram of how relevant studies were identified. A total of 12875 articles were identified through the PubMed database search and an additional 3050 articles were retrieved through other sources (additional databases and hand search of relevant bibliographies). After duplicates were removed there were 2185 potentially-relevant articles. In all, 2103 studies were excluded during the initial screening through review of titles and abstracts in addition to 14 foreign language studies. The full texts of the remaining 68 studies were thoroughly reviewed. Of these, 26 were excluded due to: randomized clinical trials (n = 7), incomplete data (n = 3), T-score report but not BMD (n = 4), overlapping reports from the same research group (n = 16). The remaining 38 studies were included in the final analysis. See supporting information file showing detailed search criteria for PubMed-MeSH database (S1 Table).

### Characteristics of studies included in the final analysis

The 38 studies included were published between 1990 and 2017: 7 came from China [11,21–26], 3 from Brazil [27–29], 3 from Spain [30–33], 2 from the United States [7,34], 2 from Germany [35,36], 2 from Hungary [37,38], 2 from Mexico [8,12], 2 from Italy [1], 2 from Sweden [4,39] and 1 each from the United Kingdom [40], South Africa [41], Australia [3], Denmark [42], Italy [1,43], Belgium [44], Norway [45], Austria [46], Bulgaria [47], Thailand [48], Singapore [49], The Netherlands [50] and Japan [51]. In total 3442 SLE cases and 6198 controls were included in the analysis of BMD (9232 women and 408 men). Eleven studies were suitable for BMD comparison in SLE patients receiving GCT (n = 358) or not (n = 354).

The study characteristics are described in Table 1 including the Oxford Centre for EBM 2011 Levels of Evidence. The most frequent level of evidence was level IV in 25 studies, followed by 8 and 3 studies corresponding to level II and III of evidence, respectively. The characteristics of studies evaluating VF are shown in Table 2. Only 5 articles evaluated VF using a standardized method, making it possible to compare BMD between SLE patients with and (n = 189) and without VF (n = 506).

**Table 1:** Characteristics of studies included comparing SLE cases and controls.

Study	Setting	Ethnicity	SLE participants				Controls				Menstrual status in SLE		GCT use in SLE (%)	GCT dose	Level of evidence
			Total	Age*	Female	Male	Total	Age*	Female	Male	Pre	Post			
Dhillon 1990	UK	NR	22	NR	22	0	14	NR	14	0	22	0	54.5	At least 10 mg/d for at least 6 months	IV
Kalla 1993	South Africa	NR	46	31	46	0	108	NR	108	0	46	0	47.8	NR	IV
Formiga 1995	Spain	Caucasian	74	30	74	0	50	NR	50	0	74	0	-	Mean cumulative dose: 32.5 (range 2.7–116.4) g	III
Pons 1995	Spain	Caucasian	43	NR	43	0	43	NR	43	0	43	0	65.1	Cumulative range dose: 2.4–68.7 g	IV
Formiga 1996	Spain	Caucasian	20	37	0	20	40	39	0	40	NR	NR	-	Cumulative dose 17.6 (range 8–60) g	IV
Chen 1996	China	Asian	56	31	56	0	15	29	15	0	56	0	98.2	Mean 10 mg/d	IV
Kipen 1997	Australia	NR	97	44	97	0	0	-	-	-	60	37	71	Median 11.6 (0.5–85.6) mg/d	IV
Hansen 1998	Denmark	NR	36	39	35	1	0	-	-	-	20	11	NR	Cumulative dose: 12.5 (3.0–22. 9) g	IV
Sinigaglia 1999	Italy	NR	84	30	84	0	145	NR	145	0	84	0	100	Cumulative dose 23.1 (0.2-127-0) g	
Teichman 1999	Germany	NR	55	58	55	0	20	54	20	0	33	22	63.6	NR	IV
Jardinet 2000	Belgium	NR	35	30	35	0	0	-	-	-	35	0	82.8	Cumulative dose 15.8 ± 14.9 g	IV
Gilboe 2000	Norway	100% Caucasian	75	45	66	9	150	NR	132	18	38	28	85	Cumulative dose 21.8 (0.23–113.4) g	IV
Redlich 2000	Austria	NR	30	33	30	0	39	NR	39	0	30	0	67	Cumulative dose 14.6 (3.5) g	IV
Becker 2001	Germany	NR	64	33	33	31	0	-	-	-	62	2	87.5	2.5–100 mg PDN/day	IV
Bhattoa 2001	Hungary	NR	23	46	0	23	40	48	0	40	-	-	91.3	Cumulative dose 33.4 (0–144.1) g	IV
Lakshminarayanan 2001	US	Caucasian	92	33	92	0	0	-	-	-	44	48	97.8	Cumulative dose 31.6 (0.5–194.9) g	IV
Bhattoa 2002	Hungary		79	49	79	0	0	-	-	-	30	49	89.7	Cumulative dose 13.8 (0–94.7) g	IV
Boyanov 2003	Bulgaria	NR	48	NR	48	0	0	-	-	-	35	13	66.6	Cumulative dose 34.4 (9–90) g	IV
Uaratanawong 2003	Thailand	Asian	118	NR	118	0	0	-	-	-	118	0	62.7	Cumulative dose 5.2 (0.14–27.0) g	IV
Coimbra 2003	Brazil	Mixed	60	33	60	0	64	31	64	0	60	0	98.3	Cumulative dose 29.7 (1.2–76.7) g	III
Borba 2005	Brazil	Mixed	70	32	70	0	20	32	20	0	70	0	NR	With VF 15.3± 12.7 mg/day and without VF 15.7±13.1 mg/d	II
Mok 2008	China	Asian	40	43	0	40	40	43	0	40	.	-	85	-	II
Li 2009	China	Asian	152	48	152	0	0	-	-	-	48	104	91.4	Median dose 6.4 (4.7–8.4) mg/d	II
Mendoza-Pinto 2009	México	Mixed	210	43	210	0	0	-	-	-	106	104	-	Mean cumulative dose 18.7 ± 19.5 g/d	II
Almehed 2010	Sweden	Caucasian	150	40	150	0	0	-	-	-	67	81	86	Cumulative dose 11 (0.1 to 97.5)	III
Alele 2011	US	Cauca	153	White: 45 Black: 42	153	0	4920	White: 53 Black: 43	4920	0	92	61	NR	NR	II
Mok 2012	China	Asian	353	42	331	22	0	-	-	-	226	127	81	Mean 4.6 ± 4.4 mg/d	II
Tang 2013	China	Asian	180	42	180	0	180	43	180	0	110	70	100	Cumulative 18.6 (10.6–27.8) g	II
Mak 2013	Singapore	Asian	45	50	37	8	45	49	37	8	13	24	NR	Cumulative dose10.00 ± 10.7 g	IV
Jacobs 2013	The Netherlands	72.2% Caucasians	126	39	113	13	0	-	-	-	103	23	51.6	Mean 9.2±10.8 mg/d	IV
Furukawa 2013	Japan	Asian	52	NR	52	0	0	-	-	-	33	19	92.3	Mean daily dose: 8.4 mg	IIb
Bonfá 2015	Brazil	NR	211	33	211	0	154	32	154	0	211	0	75.4	Cumulative dose: 28.6±22.1 g	IV
Ajeganova 2015	Sweden	NR	111	49	99	12	111	49	99	12	63	48	60.4	Median dose 5.0 (3.7–10.0) mg/d	IV
Sun 2015	China	Asian	119	33	119	0	0	-	-	-	105	14	0	0	IV
Salman-Monte	Spain	Caucasian	67	53	67	0	0	-	-	-	29	38	80.5	NR	IV
Carli 2016	Italy	Caucasian	186	46	175	11	0	-	-	-	128	58	NR	Mean cumulative dose in patients with OP 37.1 ± 25.5 g; without OP 24.4 ± 16.6 g	IV
Guo 2017	China	Asian	60	26	0	60	0	-	-	-	-	-	0	0	IV

Abbreviation: GCT = glucocorticoid therapy; d = day; g = grams; mg = milligrams; OP = osteoporosis; PDN = prednisone; NR = Not reported; UK = United Kingdom; US = United States; VF = vertebral fracture

* = mean.

**Table 2:** Characteristics of included studies comparing SLE with and without vertebral fractures.

Study	Setting	Ethnicity	SLE patients with vertebral fractures				SLE patients without vertebral fractures				Menstrual status in SLE		GCT dose	
			Total	Age*	Female	Male	Total	Age*	Female	Male	Pre	Post	With VF	Without VF
**Borba 2005**	**Brazil**	**Mixed**	**15**	**28**	**15**	**0**	**55**	**32**	**55**	**0**	**NR**	**NR**	**15.3± 12.7 mg/day**	**15.7±13.1 mg/day**
**Mendoza-Pinto 2009**	**México**	**Mixed**	**53**	**50**	**53**	**0**	**157**	**41**	**157**	**-**	**106**	**104**	**Mean cumulative dose 25.3 ± 26 g/day**	**Mean cumulative dose 17.2±18.2 g/day**
**Li 2009**	**China**	**Asian**	**31**	**55**	**31**	**0**	**121**	**46**	**121**	**-**	**48**	**104**	**Median dose 5.0 (3.1, 8.4) mg/day**	**Median dose** **6.4 (4.8, 8.2)**
**Furukawa 2013**	**Japan**	**Asian**	**26**	**40**	**26**	**0**	**26**	**50**	**26**	**-**	**33**	**19**	**7.9 ± 4.5 mg/day**	**8.9 ±5.2 mg/day**
**Bonfá 2015**	**Brazil**	**NR**	**64**	**36**	**64**	**0**	**147**	**32**	**147**	**0**	**211**	**0**	**Cumulative dose** **28.8±20.8 g**	**Cumulative dose** **28.5±22.7 g**

GCT = glucocorticoid therapy; g = grams; mg = milligrams; VF = vertebral fracture

* = mean

NR = Not reported

### Results of meta-regressions

In the three groups analyzed, we found that the best variance structure was a two level model with random effects at the level of single effect measures and another random effect at the level of studies. In addition, we assessed the need for adjustment by gender and found no significant effect for this variable in any group. The only covariate that needed adjustment was the region of the measure, which appears systematically in the three final models. Table 3 shows the most relevant results from this analysis. High heterogeneity (see I2 in Table 3) between studies was observed.

**Table 3:** Results of the most parsimonious models for the groups of studies.

Group	Mean structure	estimate	se	ci.lb	ci.ub	Pval	
Case-Controls	intrcpt	1.0023	0.0129	0.9771	1.0275	< .0001	I2
	Region2	-0.1842	0.0065	-0.197	-0.1714	< .0001	97.5054
	Region6	-0.1052	0.0074	-0.1196	-0.0907	< .0001	R2
	Individual type CTR	0.0566	0.0069	0.0431	0.0701	< .0001	0.5229
GTC treatment	intrcpt	0.9924	0.032	0.9297	1.055	< .0001	I2
	Region2	-0.1475	0.0214	-0.1893	-0.1056	< .0001	96.8124
	Region6	-0.0855	0.0295	-0.1434	-0.0276	0.0038	R2
	treatment-	0.0259	0.0196	-0.0124	0.0643	0.1855	0.2623
Vertebral fractures	intrcpt	0.9204	0.0357	0.8504	0.9904	< .0001	I2
	Region2	-0.1592	0.0203	-0.1989	-0.1194	< .0001	95.7399
	Region6	-0.0777	0.0152	-0.1075	-0.0479	< .0001	R2
	FV-	0.0331	0.0137	0.0063	0.06	0.0156	0.2621

Reference categories for Region, case-control status, treatment status and vertebral fractures status are Region 1, Case, GCT treatment + and FV + respectively. Region1: Lumbar, Region 2: Femoral, Region 6: total hip. Individual type Case: cases, Individual type CTR: controls. GCT treatment+: group under treatment; GCT treatment-: group without treatment. FV+: group with fractures; FV: group without fractures. I2: Heterogeneity by means of Higgins’s tests

Abbreviation: GCT: glucocorticoid; ci.lb: confidence interval lower bound; ci.ub: confidence interval upper bound

Evidence of significant publication bias was identified by means of the rank correlation test for funnel plot in the case-control analysis group (p-value = 0.0446), but was not observed in the other two groups (S1 Fig.).

### Comparison between SLE patients and controls

We identified 8986 participants (2899 patients with SLE vs. 6087 controls) in the analysis of BMD. There were significant differences in mean BMD between cases and controls, with controls having a higher BMD (mean difference cases/controls: -0.0566 95% CI (-0.071, -0.0439; p = < 0.0001). S2 Fig shows the forest plot corresponding to this meta-regression.

Comparison of BMD between SLE patients with (n = 348) and without (n = 169) GCT therapy showed no significant differences in BMD (S3 Fig) between treated and non-treated SLE patients (p-value: 0.1303).

Comparison of BMD between SLE patients with and without VF 694 SLE patients were included in the analysis of VF (189 with VF vs. 505 without VF) (Table 2). Patients with VF had a lower BMD than those without (mean difference without FV–with FV: 0.033; 95%CI: (0,006–0.06); p-value: 0.0156). S4 Fig shows the forest plot corresponding to this analysis.

## Discussion

This study provides insights into the inconsistently reported relationship between SLE and BMD through a meta-analysis implementing a meta-regression tool. As suggested by the majority of studies reviewed studies our meta-analysis concluded that, overall, individuals with SLE have a lower BMD than non-SLE controls. Similarly, a recent meta-analysis showed that SLE patients had significantly lower BMD levels than controls in the whole body, femoral neck, lumbar spine and total hip [14]. The strength of this meta-analysis using a meta-regression lies in evaluating the anatomical regions of BMD measure as a whole, acting as covariates in the regression, without the need for subgroup analyses that might have a greater bias as the previous meta-analysis did [14].

No gender-specific differences were found between SLE cases and controls, even though low BMD is a gender-related condition. Both female and male SLE patients had a lower BMD at any region (lumbar spine, total hip and femoral neck) than healthy controls. To date, no gender-specific differences have been reported in these pathogenic mechanisms, although an already-fragile bone, such as that observed in postmenopausal women due to hormonal loss, may play a role in accelerating bone structure disruption [52]. Most studies included involved a higher number of premenopausal (n = 1913) than postmenopausal women (n = 823), which could be a reason for the lack of impact of gender on BMD.

We found no significant differences in the effect of GCT therapy on BMD at different regions between SLE patients with and without GCT. GCT are widely used to treat SLE disease flares and complications and might have beneficial effects by reducing the adverse effects of systemic inflammation on bone. The beneficial effects produced by suppressing the impact of inflammation on bone turnover might outweigh the harmful effects of GCT. Cross-sectional studies on the relationship between glucocorticoid use and BMD in SLE show conflicting results [53]. There is a wide disparity in the GCT doses, range, mean and cumulative dose and time of exposure in the studies evaluated that could partly explain the lack of between-group differences and result in probable bias [54].

The pathophysiologic mechanisms of impaired bone quality in GCT users remain unclear. Bone quality is determined by architecture, turnover, microdamage accumulation, mineralization and bone matrix protein such as collagen. In vivo studies have shown that GCT administration causes low bone turnover due to the suppression of osteoblast function, the induction of apoptosis in osteoblasts [55], and a major loss of trabecular connectivity [56]. GCT also affect bone geometry by reducing bone formation in periosteal surfaces [57]. This suggests that GCT administration leads to deterioration in bone structure. Since very few studies have assessed the trabecular microstructure either by computed tomography (CT) or magnetic resonance techniques in SLE [58–61], these studies were not included in our meta-analysis.

VF are the most common type of osteoporotic fracture and are associated with substantial morbidity and decreased survival. They are diagnosed using the Genant semi-quantitative method, which requires a ≥20% decrease in vertebral height (anterior, mid or posterior dimensions), estimated visually, to diagnose a vertebral fracture. A recent meta-analysis including two studies [62,63] reported an almost three-fold higher risk of VF in SLE patients (RR 2.97, 95% CI 1.71–5.16, P < 0.001) compared with healthy controls [14]. However, in the studies included, VF were not assessed using a standardized method such as the Genant semi-quantitative method and, in addition, the impact of BMD measurements was not evaluated. In our meta-analysis, including more studies (five)[11,12,28,29,51] using a standardized method for VF detection, SLE patients with VF had lower BMD measurements either at the lumbar spine, total hip or femoral neck, compared to patients without VF, even though VF in GCT-induced osteoporosis may occur at higher BMD measurements than those associated with postmenopausal osteoporosis, according to a large study [64]. As mentioned, we found no significant effect for gender in any group, including VF. We were not able to evaluate the direct impact of GCT use on the VF risk, because not all studies included measured this relationship and also because differences in reporting steroid use limited the ability to extract this information.

Meta-regression is a tool used in meta-analysis to examine the impact of moderator variables on study effect sizes using regression-based techniques. This type of approach was different than that used by a previous meta-analysis [14] which used subgroup analyses techniques. Meta-regression is more effective at this task than standard meta-analytic techniques [65].

Our study has several limitations and the results should be interpreted with caution. First, the review is preliminary, partly due to the scarcity of reports, making definitive conclusions difficult. We included only studies in adults and did not include children or adolescents because bone density differs considerably between them. Secondly, there was high heterogeneity (see Table 3) between studies. A meta-analysis using individual patient data is recommended to provide good evidence for bone loss and fracture risk in SLE patients. Additionally, there were variations in the definition of GCT therapy between studies. Several methods were identified to define the risk of low BMD due to GCT treatment in SLE patients. However, we used “current use” or “ever use” binary response definitions to compare SLE patients with or without GCT therapy. The “current use” definition examines the association between BMD or fracture and whether the patient was exposed to GCT on the day of the measurements: important assumptions for this definition are that any prior GCT exposure does not affect the risk of low BMD or fracture, and the dose of GCT on the day of the measurements is not important. The “ever use” definition, conversely, assumes that all historical therapy affects the risk of fracture, but is regardless of how recently the therapy was taken. Thirdly, several covariates of clinical importance were not included in our analysis, such as ethnicity and post-menopausal status when comparing SLE patients and controls. Fourthly, we were not able to calculate odd ratios or relative risk for VF in SLE patients, since most studies in this subgroup analysis did not include healthy controls. Fifthly, most studies included had cross-sectional designs with a small sample size; there is no doubt that large prospective cohort studies adjusting for cofounders are more appropriate in assessing the fracture risk in SLE patients, in order to establish a real temporal cause-effect relationship. In fact, our review has disclosed the scarcity of longitudinal studies on this topic. We believe this meta-regression should encourage the research community to initiate and design future cohort studies.

Finally, significant publication bias was found for the first analysis (comparison of SLE patients and controls). However, this may probably be due to the small study effect rather than true publication bias, especially in the presence of significant heterogeneity between studies [66]. The majority of meta-analyses are based on a series of studies to produce a point estimate of an effect and measures of the precision of that estimate. In addition, a meta-regression model seeks to determine whether a study-level covariate is a plausible source of heterogeneity in a set of treatments or an output variable effect. Upon doing such analyses, as we have shown in the present study, the bias of the small study sample size of the independent studies is overcome. As studies become less precise, such as in smaller trials, the results of the studies can be expected to be more variable than the more precise larger studies; this aspect was cancelled out in the present study through the publication bias analyses and, as a result, allowed an objective assessment[67,68].

In conclusion, this meta-analysis, using meta-regression analytic techniques, showed that SLE patients have lower BMD levels than healthy controls independently of the skeletal site of measurement and gender. In addition, BMD levels at any region did not differ between SLE patients with and without GCT therapy. SLE patients with VF have lower BMD levels than those without. Larger prospective cohort studies are needed to provide a more accurate assessment of the relationship between SLE and fracture risk. Future studies evaluating effective osteoporosis screening and prevention in SLE patients are also required.

## Supporting information

S1 Fig Funnel plots for the three models supplementary. A. Funnel plot of the model for case-controls. B. Funnel plot for GCT therapy. C. Funnel plot for vertebral fractures.
(TIF)

S2 Fig Forest plot for case- control study. Regions codified are as follows: 1) lumbar spine 2) femoral neck and 3) total hip.
(TIF)

S3 Fig Forest plot for therapy effect study. Regions codified are as follows: 1) lumbar spine 2) femoral neck and 3) total hip.
(TIF)

S4 Fig Forest plot for vertebral fractures study. Regions codified are as follows: 1) lumbar spine 2) femoral neck and 3) total hip.
(TIF)

S1 File PRISMA checklist. (PDF)

S1 Table Detailed search criteria for PubMed-MeSH database. (PDF)

S2 Table Glucocorticoid use definitions for the analysis comparing patients with and without GCT therapy. (PDF)
