# Supplementary figures and images for: Bone mineral density and vertebral fractures in patients with systemic lupus erythematosus: A systematic review and meta-regression
Source: PLoS One. 2018 Jun 13;13(6):e0196113. doi: 10.1371/journal.pone.0196113 (PMC5999233; doi:10.1371/journal.pone.0196113)

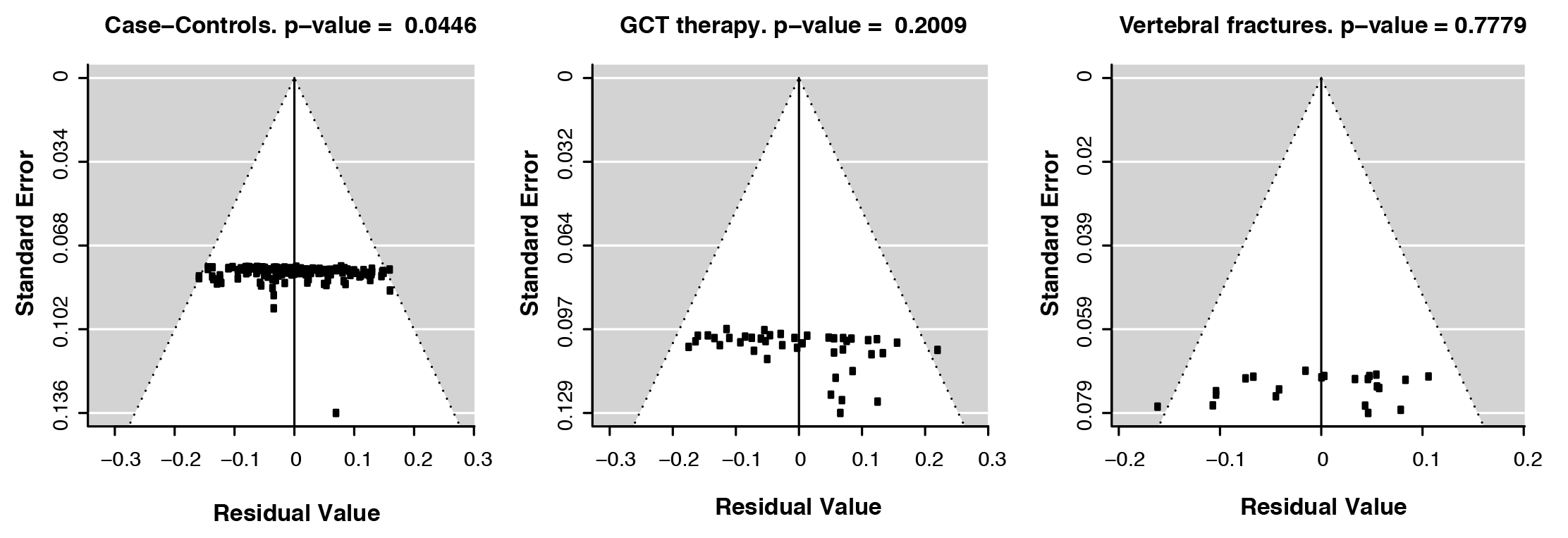

Supplement: S1 Fig — A. Funnel plot of the model for case-controls. B. Funnel plot for GCT therapy. C. Funnel plot for vertebral fractures. (TIF) [file pone.0196113.s001.tif]

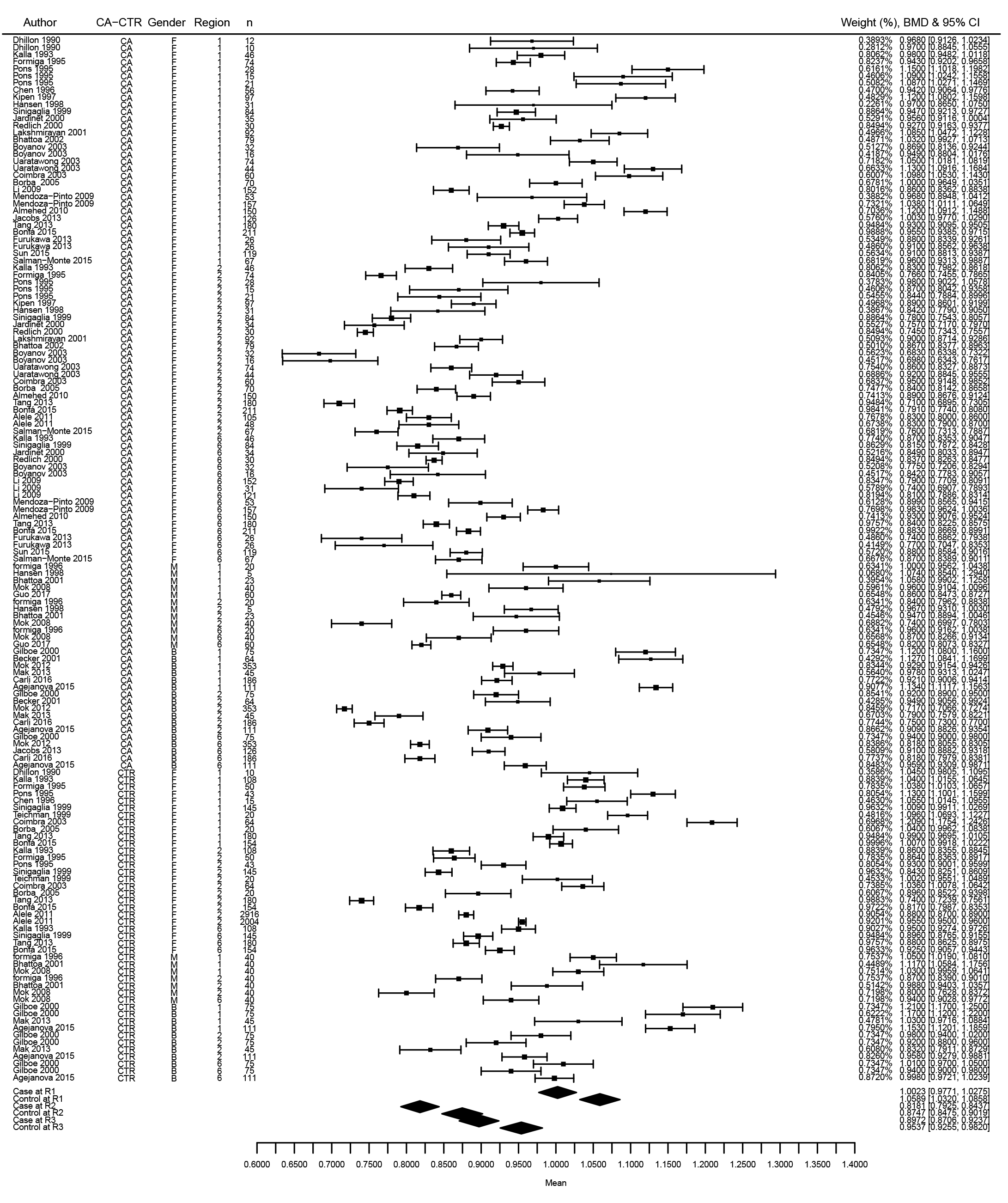

Supplement: S2 Fig — Regions codified are as follows: 1) lumbar spine 2) femoral neck and 3) total hip. (TIF) [file pone.0196113.s002.tif]

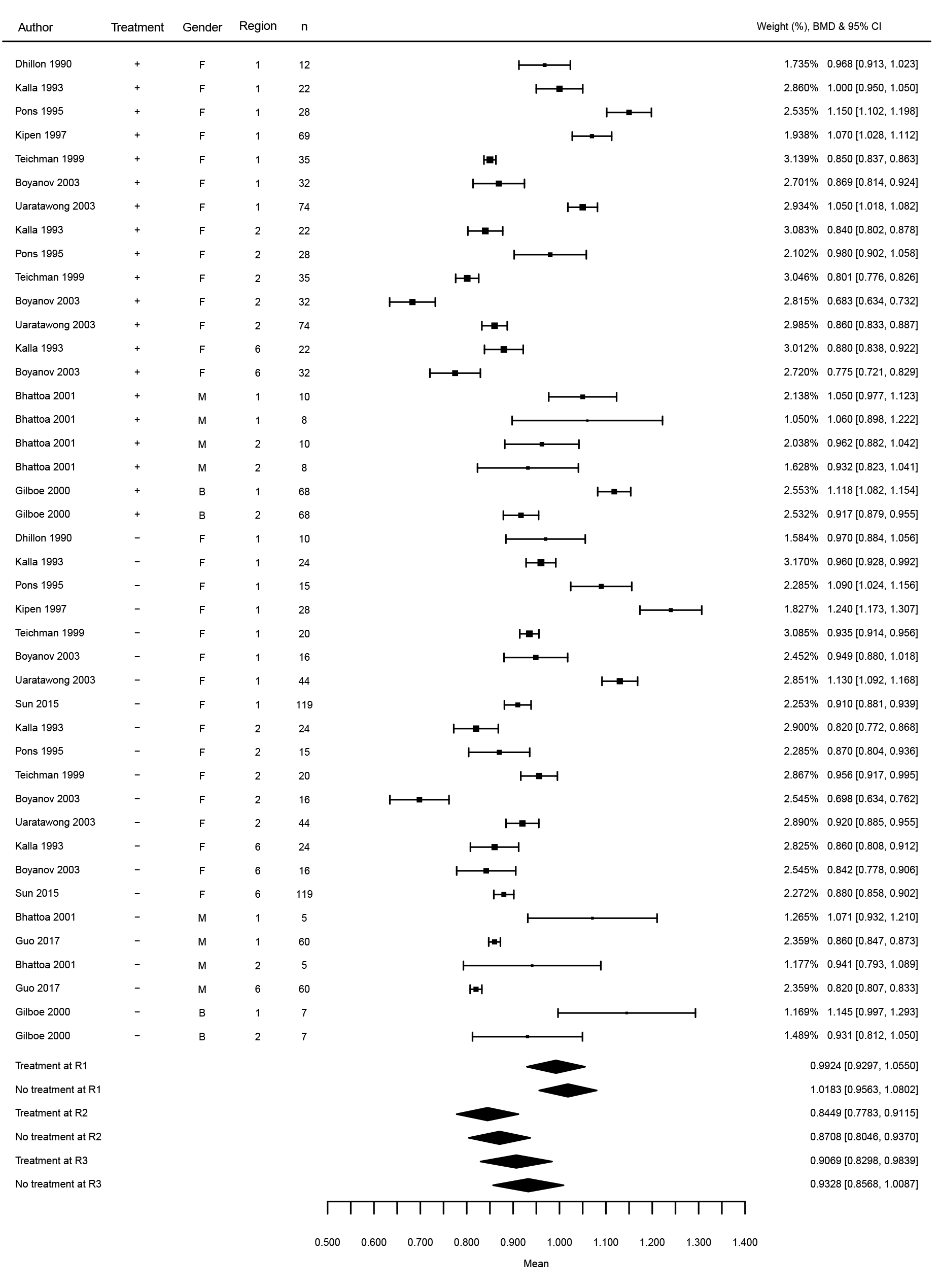

Supplement: S3 Fig — Regions codified are as follows: 1) lumbar spine 2) femoral neck and 3) total hip. (TIF) [file pone.0196113.s003.tif]

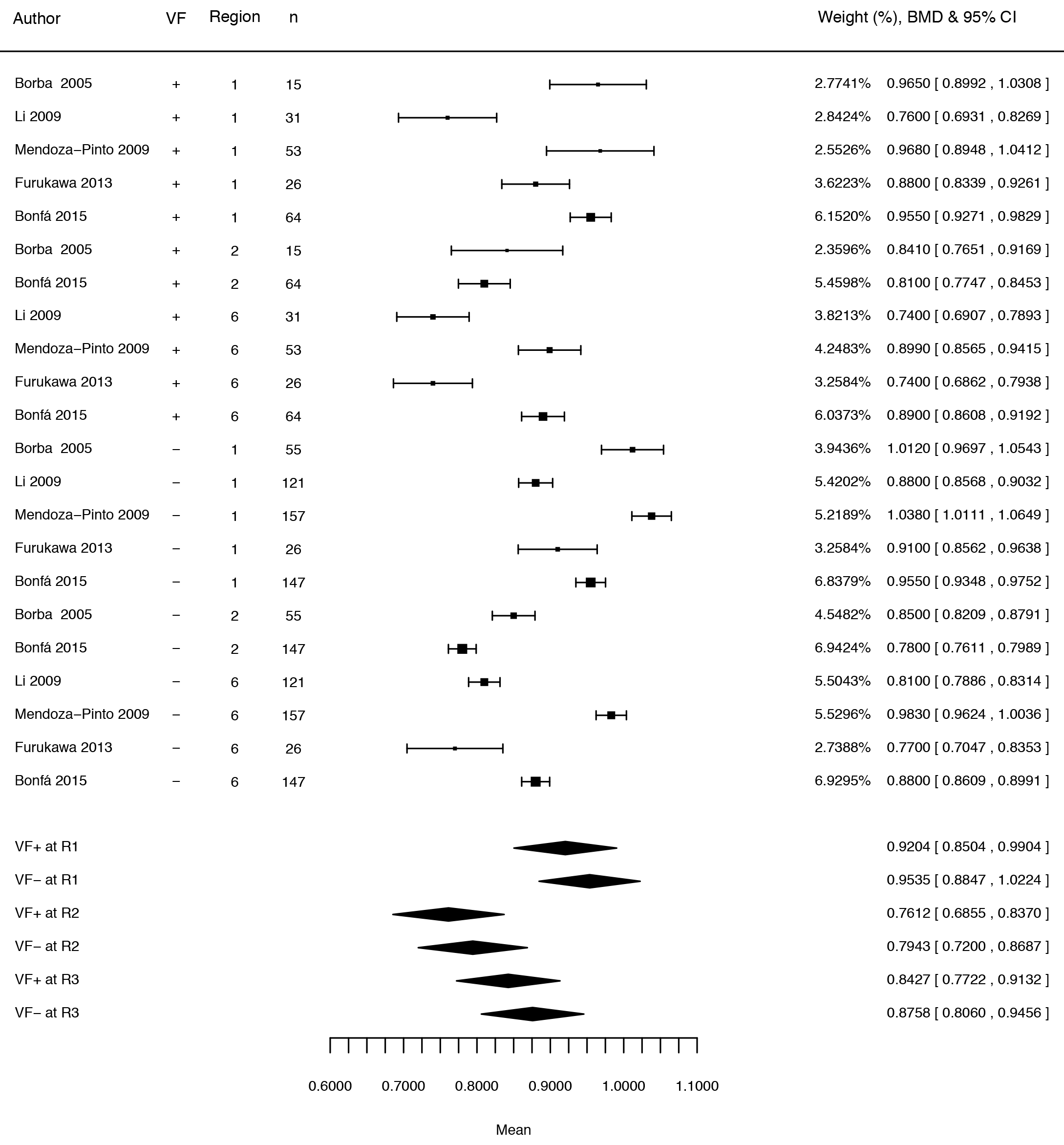

Supplement: S4 Fig — Regions codified are as follows: 1) lumbar spine 2) femoral neck and 3) total hip. (TIF) [file pone.0196113.s004.tif]
