# Supplementary material for: Bone mineral density and vertebral fractures in patients with systemic lupus erythematosus: A systematic review and meta-regression
Source: PLoS One. 2018 Jun 13;13(6):e0196113. doi: 10.1371/journal.pone.0196113 (PMC5999233; doi:10.1371/journal.pone.0196113)
Supplement: S1 Table — (PDF) [file pone.0196113.s006.pdf]

S1 Table

The following Medical Subject Heading (MeSH) terms were implemented in PubMed database:<br>• "Lupus Erythematosus, Systemic"<br>• "Osteoporosis"<br>• "Bone Density"<br>• "Densitometry"<br>• "Spinal Fractures"
We applied the following Boolean operators to develop the search: "AND" and "OR" retrieving different MeSH Heading/Subheading Combination<br>The following combinations and the corresponding number of studies were retrieved:<br>• (((("Lupus Erythematosus, Systemic"[Mesh]) AND "Osteoporosis"[Mesh]) OR "Bone Density"[Mesh]) OR "Densitometry"[Mesh]) OR "Spinal Fractures"[Mesh] = 77816<br>• (("Lupus Erythematosus, Systemic"[Mesh]) AND "Osteoporosis"[Mesh]) OR "Bone Density"[Mesh] = 47576<br>• (((("Lupus Erythematosus, Systemic"[Mesh]) AND "Osteoporosis"[Mesh]) AND "Bone Density"[Mesh]) OR "Densitometry"[Mesh]) OR "Spinal Fractures"[Mesh] = 44429<br>• (((("Lupus Erythematosus, Systemic"[Mesh]) AND "Osteoporosis"[Mesh]) AND "Bone Density"[Mesh]) OR "Densitometry"[Mesh]) = 32167<br>• (((("Lupus Erythematosus, Systemic"[Mesh]) AND "Osteoporosis"[Mesh]) OR "Bone Density"[Mesh]) OR "Densitometry"[Mesh]) = 66812<br>• (((("Lupus Erythematosus, Systemic"[Mesh]) AND "Osteoporosis"[Mesh]) OR "Bone Density"[Mesh]) AND "Densitometry"[Mesh]) = 12875<br><br>We consider the last Boolean and MeSH terms search had the best performance. The following steps in the systematic review are detailed in Figure 1. (Flow diagram detailing the literature search)
