# Supplementary material for: Bone mineral density and vertebral fractures in patients with systemic lupus erythematosus: A systematic review and meta-regression
Source: PLoS One. 2018 Jun 13;13(6):e0196113. doi: 10.1371/journal.pone.0196113 (PMC5999233; doi:10.1371/journal.pone.0196113)
Supplement: S2 Table — (PDF) [file pone.0196113.s007.pdf]

S2 Table

	Without GCT	With GCT
Dhillon 1990	Never user 10	At least 10 mg a day for at least 6 months 12
Kalla 1993	Current user but had only begun such treatment within the previous 6 months 24	Current corticosteroid for longer than 6 months prior study 22
Pons 1995	Never user 15	Ever user 28
Kipen 1997	Never user 28	Ever user 69
Teichman 1999	Never user 20	Ever user 35
Gilboe 2000	Never user 7	Ever user 68
Bhattoa 2001	Current no-user 5	Current daily steroid dose > 7.5 mg/day 8<br>Current daily steroid dose ≤ 7.5 mg/day 10
Boyanov 2003	Never user 16	Ever user 32
Uarantanawong 2003	Never user 44	Ever user 74
Sun 2015	Never user 119	Ever user 0
Guo 2017	Never user 60	Ever user 0
